# Relationship between Nutrition, Lifestyle Habits and Laboratory Parameters in Hypertensive Patients with/without Cognitive Dysfunction

**DOI:** 10.3390/life13020311

**Published:** 2023-01-22

**Authors:** Kinga-Ilona Nyulas, Márta Germán-Salló, Zita Fazakas, Zoltán Preg, Tünde Pál, Sándor Pál, Robert Gabriel Tripon, Margit Judit Cseh, Zsuzsánna Simon-Szabó, Emil Marian Arbănași, Enikő Nemes-Nagy

**Affiliations:** 1 Doctoral School of GE Palade University of Medicine, Pharmacy, Science, and Technology of Târgu Mureş, 540136 Târgu Mureş, Romania; 2 Department of Internal Medicine II, Faculty of Medicine, GE Palade University of Medicine, Pharmacy, Science, and Technology of Târgu Mureş, 540136 Târgu Mureş, Romania; 3 Department of Biochemistry and Chemistry of Environmental Factors, Faculty of Pharmacy, GE Palade University of Medicine, Pharmacy, Science, and Technology of Târgu Mureş, 540136 Târgu Mureş, Romania; 4 Department of General Medicine, Faculty of Medicine, GE Palade University of Medicine, Pharmacy, Science, and Technology of Târgu Mureş, 540136 Târgu Mureş, Romania; 5 Emergency Institute for Cardiovascular Diseases and Transplantation, GE Palade University of Medicine, Pharmacy, Science, and Technology of Târgu Mureş, 540136 Târgu Mureş, Romania; 6 Department of Laboratory Medicine, Department of Transfusion Medicine, Medical School, University of Pécs, H-7622 Pécs, Hungary; 7 Department of Ophthalmology, Faculty of Medicine, GE Palade University of Medicine, Pharmacy, Science, and Technology of Târgu Mureş, 540136 Târgu Mureş, Romania; 8 Nutrition and Dietetics Deparment, GE Palade University of Medicine, Pharmacy, Science, and Technology of Târgu Mureş, 540136 Târgu Mureş, Romania; 9 Department of Pathophysiology, Faculty of Medicine, GE Palade University of Medicine, Pharmacy, Science, and Technology of Târgu Mureş, 540136 Târgu Mureş, Romania; 10 Clinic of Vascular Surgery, Mureș County Emergency Hospital, 540136 Târgu Mureș, Romania; 11 Department of Chemistry and Medical Biochemistry, Faculty of Medicine in English, GE Palade University of Medicine, Pharmacy, Science, and Technology of Târgu Mureş, 540136 Târgu Mureş, Romania

**Keywords:** hypertension, nutrition, cognitive dysfunction, diabetes mellitus, laboratory parameters

## Abstract

**Simple Summary:**

The severity of hypertension is correlated with cognitive dysfunction. Nutritional factors have a major contribution to the state of health. This study proposed to evaluate nutrition and lifestyle habits in hypertensive patients with/without cognitive dysfunction and to establish correlations to laboratory parameters. Our results showed a high incidence of diabetes mellitus and glucose intolerance as a comorbidity in our subjects, with zinc deficiency found in 74% of the subjects. Microalbuminuria was significantly higher in the subgroup with cognitive dysfunction. The daily intake of magnesium and cholesterol was significantly lower in patients with cognitive dysfunction compared to patients without cognitive dysfunction. The incidence of high body weight and obesity was high in the studied groups. Data provided by our research can contribute to the better management of hypertensive patients, revealing improper dietary habits, deficiencies and cardiovascular risk factors.

**Abstract:**

(1) Background: Cognitive dysfunction is a major concern in hypertensive patients. Lifestyle habits and nutrition influence laboratory parameters, with an impact on clinical course. The objective of the study was to evaluate nutrition and lifestyle habits in hypertensive patients with/without cognitive dysfunction and establish correlations to laboratory parameters. Material and Methods: 50 patients admitted to the Cardiovascular Rehabilitation Clinic in Târgu Mureș were enrolled in this study between March–June 2021. We evaluated their cognitive function, and they filled in a questionnaire about lifestyle and nutrition. Biochemical blood tests were performed using a Konelab Prime 60i analyzer. IBM-SPSS22 and GraphPad InStat3 were used for statistics. Results: Mean age of hypertensive patients (n = 50) was 70.42 ± 4.82 (SD) years, half of them had cognitive dysfunction. Zinc deficiency was present in 74% of the subjects. The subgroup with cognitive dysfunction had significantly higher BMI (*p* = 0.009) and microalbuminuria (*p* = 0.0479), as well as significantly lower magnesium intake (*p* = 0.032) and cholesterol intake (*p* = 0.022), compared to those with normal cognitive status. Conclusions: Nutrition is in a close relationship with laboratory parameters; significant differences (microalbuminuria, cholesterol intake, BMI, etc.) are present between hypertensive patients with/without cognitive dysfunction. A healthy diet is important for the maintenance of metabolic balance, the achievement of optimal body weight, and the prevention of complications.

## 1. Introduction

Arterial hypertension (HT) is one of the most frequent chronic diseases, with increasing prevalence. Almost one third of the global population is affected. In the Romanian population, the prevalence is 45% and it shows an increasing incidence [1,2,3]. HT and its complications account for 62% of all deaths in Romania. According to a recent national study, the SEPHAR III survey, in 2016 there were approximately 7.4 million high blood pressure (HBP) patients and 1.8–1.9 million high normal blood pressure (HNBP) adult subjects in Romania. Risk factors for HNBP and HBP were DM and dyslipidemia, as well as being overweight or obese. Depression was also a risk factor for HBP, but it was not associated with HNBP. Daily alcohol consumption (300 mL wine or 30 mL strong drinks) was found to non-significantly increase the risk of HNBP and HBP. Cigarette smoking had negative association with HBP and was not associated with HNBP. Salt intake was significantly associated with HNBP and HBP, regardless of age or sex [4,5].

According to World Health Organization’s (WHO) data, 1.28 billion adults globally aged 30–79 have high or elevated blood pressure, and 46% of them are unaware of their condition [6,7].

High blood pressure, especially if untreated, is associated with elevated risk of cognitive dysfunction, as well as lower cognitive performance in elderly patients [8].

Inadequately treated hypertension compromises the structural and functional integrity of the cerebral microcirculation, promoting microvascular rarefaction, neurovascular dysfunction, blood–brain barrier disruption, neuroinflammation, cerebral microhemorrhages, lacunar infarction and white matter damage. All these microvascular complications aggravate cognitive decline [9].

Previous studies have demonstrated an indisputable relationship between arterial hypertension and cardiovascular diseases, such as ischemic heart disease and heart failure [10,11], but it is also a major risk factor for stroke [12]. It can be considered a modifiable risk factor with proper blood pressure control, healthy lifestyle and/or antihypertensive medication. Despite all efforts in blood pressure control, results are insufficient and high blood pressure still represents a challenge in the reduction in cardiac and cerebrovascular morbidity and mortality [6,13].

The severity of hypertension is positively correlated with cognitive dysfunction, and the incidence of dementia is rising with age [14]. Cognitive dysfunction is an intermediate state between normal ageing and dementia. As the population ages, its prevalence increases together with the increase in the burden on society and economy [15].

The synergistically negative effects of hypertension and ageing, and the impaired cellular stress tolerance will lead to exacerbated adverse cerebro-microvascular effects of hypertension [9]. Cerebral microvasculopathy contributes to impaired cognitive performance in hypertensive patients [16]. 

Several screening instruments have been developed and validated for the assessment of cognitive dysfunction. During the evaluation, patients are asked to perform different tasks and complete standard questionnaires, which evaluate different cognitive domains [17].

The primarily affected cognitive functions are processing speed, working memory, short-term memory, learning and delayed recall [8]. 

Twelve potentially modifiable risk factors were identified in the development of cognitive dysfunction. In addition to arterial hypertension and diabetes, smoking, obesity, physical inactivity, depression, hearing problems, low educational and social status, air pollution, excessive alcohol intake and traumatic brain injury were also identified as risk factors for cognitive dysfunction [15].

Control of cardiovascular risk factors in middle-aged patients plays a key role in the prevention of dementia in elderly people [18]. The correction of the identified and modifiable factors might prevent cognitive impairment and dementia in up to 40% of the patients [15].

Diabetes mellitus (DM) is a chronic metabolic disorder associated with cognitive impairment, representing a major concern for public health worldwide [19]. Evidence suggests the mixed etiology of cognitive impairment in diabetic patients, due to complications of metabolic dysregulation. Cerebral hypoperfusion, cerebral ischemia, stroke, uncontrolled hyperglycemia, hypoglycemia, microvascular complications, hyperlipidemia and obesity were suggested as the promoters of the development of vascular cognitive impairment in diabetic patients [20].

It has been found that older age, prolonged duration of the disease and male gender are associated with higher risk of poor metabolic control and complications [21]. Sedentarism and unhealthy diet, as major risk factors of type 2 DM [22], lead to being overweight or obese, also favoring chronic inflammation, negatively influencing metabolic laboratory parameters, such as lipid profile and carbohydrate balance [6,23].

Literature data suggests the earlier onset of cognitive dysfunction and increased risk of dementia in the presence of DM, leading to a two-fold increase in the incidence of cognitive impairment. Moreover, diabetic patients with cognitive disorders have greater severity of both conditions, with a profound impact on quality of life, which constitutes an additional significant socio-economic burden [24,25].

Dietary habits contribute to the development of numerous diseases. The effect of different diets, such as the Mediterranean diet, on cognitive function was the subject of multiple studies, and good results were demonstrated in the decrease in cognitive dysfunction [26].

Nutritional factors play an essential role in health preservation of the general population. Excessive calories, particularly from a high-fat diet, induces hippocampal deficits, and leads to inflammation in the central nervous system (CNS) and subsequent cognitive decline [27]. Inadequate nutrition is not limited to the high intake of unhealthy nutrients, as the most emergent problems currently affecting at least a billion people worldwide are linked to the insufficient intake of several minerals and trace elements, such as selenium, zinc, iodine, calcium, magnesium or iron. [28].

A diet rich in antioxidants, vitamins and minerals can be beneficial in primary and secondary prevention of these abnormal conditions, including obesity, hypertension and DM. A study conducted in the Netherlands, with a follow-up for 10 years, including 37,846 men and women, confirmed that high intake of α-carotene and β-carotene decreases the risk of type 2 DM among healthy subjects in both genders [29]. Trace element deficiency, such as chromium, zinc and copper, can lead to the development of hypertension [30], DM and their complications. Evidence provided by the VITACOG trial showed that vitamin B deficiencies contribute to brain atrophy and cognitive decline in patients with mild cognitive impairment, while vitamin B supplementation diminishes the decline in the executive function and rate of global brain atrophy [31].

Oxidative stress is a significant causative factor of chronic diseases, including impaired cognitive function [27,32].

The aim of this study was to evaluate hypertensive patients based on laboratory parameters, their diet, lifestyle habits and their cognitive function (with or without cognitive dysfunction). The study focused on the relationship between metabolic parameters and diet and the differences between the two subgroups (with or without cognitive dysfunction).

## 2. Materials and Methods

### 2.1. Study Population and Design

This prospective, cross-sectional study included 50 elderly subjects admitted to the Cardiovascular Rehabilitation Clinic in Târgu Mureș, between March–June 2021. The study was approved by the Ethics Committee of the Clinical County Hospital (no. 16326/01.07.2020) and that of the “George Emil Palade” University of Medicine, Pharmacy, Science, and Technology of Târgu Mureș (nr. 1065/20.07.2020). Prior to the enrollment in the study, the informed consent document was signed by the patients.

The inclusion criteria were being aged between 59–79 years, documented grade 2 or 3 hypertension, admission to the Cardiovascular Rehabilitation Clinic in Târgu Mureș and willingness to participate.

The exclusion criteria comprised grade 1 hypertension, depression, organ failure, chronic kidney disease, acute coronary syndrome, Alzheimer’s disease, Parkinson’s disease, type 1 DM and severe metabolic imbalance. A short 13-item form of Beck Depression Inventory was used; patients with scores over 13 were excluded. Some of the patients were already diagnosed with depression; they were not included in the study.

The included subjects were dichotomized based on the presence or absence of cognitive dysfunction. The Montreal Cognitive Assessment (MOCA) test was used to assess the patients’ cognitive function; a score under 26 was considered positive for cognitive dysfunction.

### 2.2. Data Collection and Paraclinical Tests

The authors collected demographic and anamnestic data and determined the subjects’ body mass index (BMI). To assess lifestyle and diet over the previous one-year period, patients completed validated questionnaires. The MOCA test has also been administered for cognitive status assessment, and Beck’s Depression Inventory test of depression screening was used as well to exclude depression.

Blood samples were also collected from the patients using vacutainers containing clot activator for measurement of metabolic parameters, and other vacutainers containing 3.8% sodium citrate for erythrocyte sedimentation rate (ESR) and fibrinogen measurement (Technoclone, Vienna, Austria). Additionally, urine was collected every 24 h to evaluate microalbuminuria by turbidimetry. 

After centrifugation at 8000 rpm for 5 min and separation, serum samples were used for biochemical testing (serum uric acid, glucose, total and HDL-cholesterol, triglycerides, creatinine, cystatin C, zinc) on a Konelab Prime 60i analyzer (Thermo Fisher Scientific Inc, Waltham, MA, USA). Serum samples for cystatin C and zinc measurement were stored in 2 mL cryotubes (IMEC SA, Bucharest, Romania) at −70 °C before being processed. Most reagents were acquired from Diagnosticum Zrt, Budapest, Hungary, except for the cystatin C (Thermo Fisher Scientific Oy, Vantaa, Finland) and zinc (Sentinel Diagnostics, Milan, Italy) reagents. LDL-cholesterol concentration was calculated using the Friedewald formula. Fibrinogen measurement was performed on a Thrombolyzer-XR equipment using Clauss assay (Behnk Electronic GmbH & Co, Norderstedt, Germany), with reagents from Technoclone, Vienna, Austria.

### 2.3. Statistics

The obtained data were introduced in a database using Microsoft Office ExcelTM, the statistical analysis was performed using IBM-SPSS, version 22 (SPSS, Inc., Chicago, IL, USA) and GraphPad InStat, version 3 software GraphPad Software Inc., San Diego, CA, USA), using unpaired Students’ *t*-test with and without Welch correction, Pearson correlation, and Fisher’s exact test. For Gaussian data distribution assessment, the Kolmogorov–Smirnov normality test was used. The threshold for statistical significance was set at *p* < 0.05.

## 3. Results

### 3.1. Demographic Data Results

The average duration of hypertension was 18.08 ± 12.14 (SD) years. Based on their MOCA test result, the hypertensive patients were divided into two subgroups: hypertensives with cognitive dysfunction (score under 26 of 30) and without cognitive dysfunction (score above 26). 

The average age of the hypertensive patients in the group with cognitive dysfunction was 70.96 ± 5.65 (SD) years, and the difference is not significant (*p* = 0.434) compared to the group of hypertensive patients without cognitive dysfunction (69.88 ± 3.84 years), using unpaired Student t-test with Welch correction.

### 3.2. Laboratory Parameters Comparison in the Study Groups

The values of the main parameters (expressed as mean ± SD) evaluated in the two groups and their statistical differences are presented in Table 1.

The average value of microalbuminuria was significantly higher (*p* = 0.0479) in hypertensive patients with cognitive dysfunction (33.25 ± 54.85 mg/24 h) compared to those with normal cognitive function (10.28 ± 4.92 mg/24 h).

### 3.3. Dietary Intake and Nutritional Status of the Subjects

Daily dietary intake of the main nutrients, fibers, water and alcohol (expressed in mean ± SD) was evaluated in the two study groups and their statistical differences are presented in Table 2. 

Dietary supplement daily intake (vitamins, minerals) expressed in mean ± SD in the study groups is represented in Table 3.

Based on data obtained from the questionnaire, fiber intake of 52% of the subjects is less than 30 g/day, 38% of subjects regularly consume small amounts of alcohol and 62% drink less than 2 L of water per day. A total of 85% of the subjects eat more than the recommended 150 mg cholesterol per day, and 49% of them have a daily cholesterol intake over 300 mg. Only 9% had higher sodium intake than the recommended 3 g/day. A total of 53% had lower zinc intake than the recommended daily dose; zinc deficiency (serum levels under 11 μmol/L) could be observed in 74% of the studied subjects.

Based on the survey results, 96% of the study groups had daily carotene intake under 12 mg, 18% had lower daily vitamin E intake and 25% presented lower vitamin C intake than recommended.

**Figure 1:**
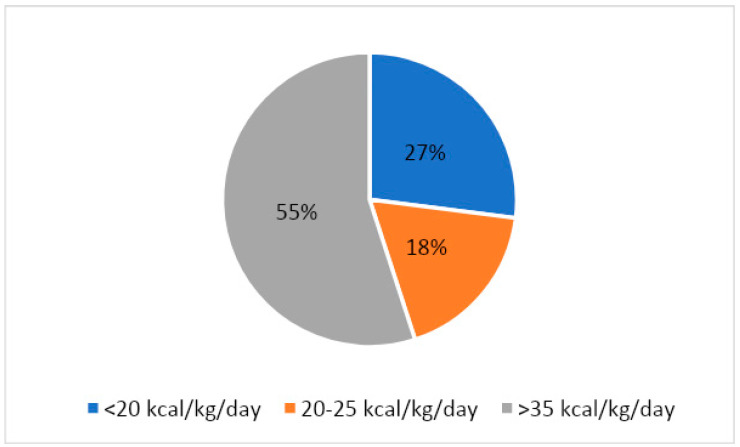
Distribution of daily energy intake in the studied hypertensive patients.

We defined the calorie intake of the patients in kcal/weight(kg)/day. The results showed that only 18% of the studied patients consume between 20–25 kcal/kg/day; 27% consume less than 20 kcal/ kg/day, which corresponds to a restrictive diet; and 55% of patients have a higher energy intake than 25 kcal/kg/day, some over 35 kcal/kg/day (27%) (Figure 1). The highest reported value was 61.84 kcal/kg/day, while the minimum calorie intake was 10.70 kcal/kg/day.

### 3.4. Correlations of Laboratory Parameters and Diet

**Figure 2:**
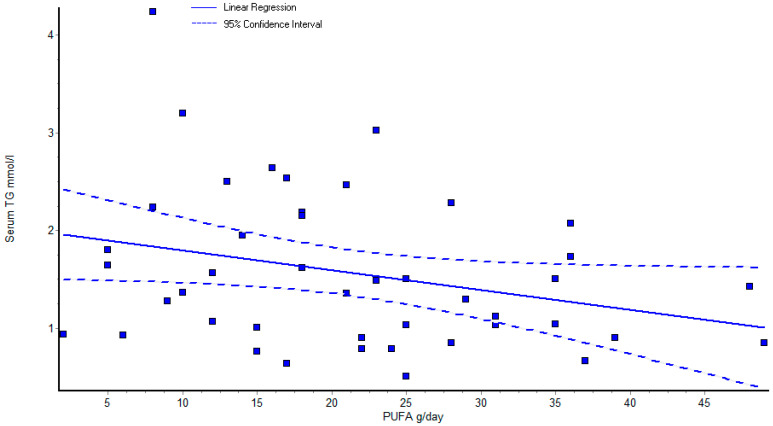
Correlation of PUFA intake with serum triglyceride levels.

The authors evaluated the correlations between laboratory parameters and daily dietary intake. A negative correlation was obtained between daily calorie intake and serum glucose levels in the studied hypertensive patients (r = −0.322, *p =* 0.031). Our results showed that uric acid presented a negative correlation with carbohydrates (r = −0.375, *p =* 0.011), fiber (r = −0.382, *p =* 0.010) and water intake (r = −0.337, *p =* 0.024) in all hypertensive patients. A negative correlation was established in serum creatinine levels and water intake (r = −0.453, *p =* 0.002), as well as PUFA (polyunsaturated fatty acid) intake with serum glucose levels (r = −0.298, *p =* 0.047), cystatin C levels with water intake (r = −0.36, *p =* 0.01), dietary fiber intake (r = −0.34, *p =* 0.02), carotene and (r = −0.29, *p =* 0.049) vitamin E (r = −0.30 *p =* 0.04) intake. PUFA intake showed negative correlation with serum triglyceride levels (r = −0.29, *p =* 0.049) (Figure 2).

We also correlated diet and inflammatory markers: fibrinogen levels had negative correlation with water intake (r = −0.32, *p =* 0.03), carotene (r = −0.35, *p =* 0.02) and vitamin B_6_ intake (r = −0.32, *p =* 0.03). HsCRP levels negatively correlated with carbohydrate intake (r = −0.35, *p =* 0.01), vitamin A (r = −0.3, *p =* 0.04), vitamin B_6_ (r = −0.29, *p =* 0.04), folic acid intake (r = −0.29, *p =* 0.04), sodium (r = −0.29, *p =* 0.04) and iron intake (r = −0.29, *p =* 0.04), and its correlation with carotene intake was not significant (r = −0.26, *p =* 0.07).

### 3.5. Distribution of Cognitive Dysfunction according to Diet

**Figure 3:**
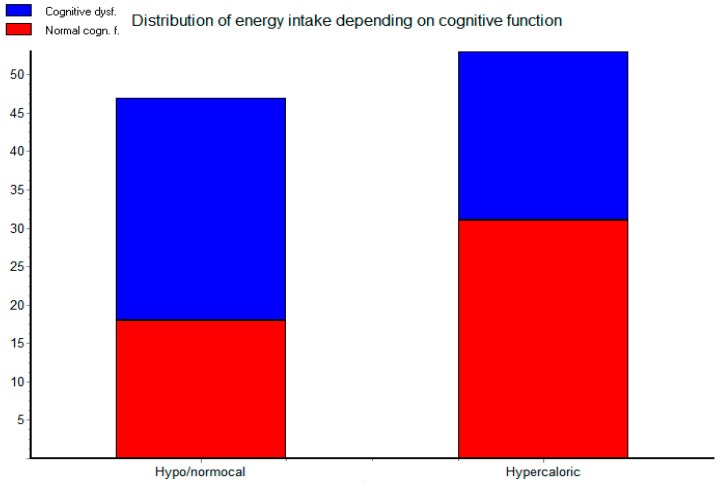
Distribution of calorie intake based on cognitive function.

The distribution of patients with and without cognitive dysfunction showed significant difference (*p =* 0.0484) between the groups on hypo/normocaloric diet and hypercaloric diet (Figure 3) using Fischer’s test. Normal cognitive function was more frequent in the group on a hypercaloric diet. 

The distribution of diabetic patients showed no significant difference between the two subgroups (*p =* 0.085), and the prevalence of diabetics was higher in the subgroup with cognitive dysfunction (56%) compared to those with normal cognitive function (28%). In addition to DM as a comorbidity, 10 of the 50 studied hypertensive subjects had glucose intolerance (20%).

## 4. Discussion

Our results show a high incidence of DM and glucose intolerance as comorbidity in our subjects and a greater risk of cardiovascular complications [33,34]. Thus, secondary prevention is particularly important in these groups, especially in the presence of cognitive dysfunction.

This study shows that hypertensive patients consume approx. 50% of the recommended daily dose of carotenoids, which are precursors for vitamin A, compared to the guidelines of the NIH. Low intake of other antioxidant vitamins (E, C) was less common in the studied group. Several studies confirmed the importance of antioxidant intake in the prevention of hypertension, DM, and their complications [35,36,37]; therefore, this should be a major focus regarding nutritional advice for these patients. High vitamin C intake can also be beneficial for its anti-inflammatory properties [38]. It is known that chronic inflammation is the basis of several chronic diseases, including atherosclerosis and DM. 

The reference ranges of the measured laboratory parameters are presented in Table 4. 

Several studies showed the association between trace element deficiency and hypertension [39]. Dietary supplements containing antioxidants are recommended [40,41], especially in the case of vitamin and mineral deficiencies, and zinc supplementation should be a major concern in our field based on the results of this study.

### Nutritional Recommendations

The US National Institute of Health’s (NIH) daily water, energy and nutrient intake recommendations are presented in Table 5 [42]. The recommended daily dietary supplement (vitamin and mineral) intake is presented in Table 6 [42].

A significantly lower magnesium intake was seen in the subgroup with cognitive impairment. Magnesium deficiency is a risk factor for atherosclerosis and endothelial dysfunction [43,44], coronary heart disease and type 2 DM [43,45]. Based on our study results, the authors consider that determining magnesium levels in all patients with these conditions should be taken into consideration. 

Serum zinc measurement is not regularly performed as a routine laboratory test in our country, but it should be recommended for patients with hypertension, especially in subjects with DM as a comorbidity. Almost three quarters of our subjects had zinc deficiency; these results are similar to those in the literature. Lower serum zinc levels and increased zinc excretion through the urine are characteristic for hypertensive patients compared to non-hypertensive subjects [46]. Metabolic syndrome is also associated with elevated elimination of urinary zinc [47,48].

Zinc deficiency can induce hypertension by enhancing sodium reabsorption [48]. Antihypertensive treatment can also be a factor causing trace element imbalance; it can influence, for example, zinc homeostasis; and zinc supplementation can decrease serum glucose levels [49]. These aspects should be taken into consideration in the treatment plan of hypertensive patients, and appropriate doses of zinc-containing dietary supplements should be administered to these subjects.

Zinc deficiency is common in several chronic diseases (including type 2 diabetes) and promotes hypertension. Experimental studies demonstrated that renal sodium transport dysregulation enhances Na^+^ reabsorption and contributes to the pathomechanism of hypertension induced by zinc deficiency [48]. Several studies showed an inverse relationship between serum zinc levels and blood pressure values. Furthermore, high blood pressure is more frequent in people with low dietary zinc intake [50].

Zinc supplementation reduces the risk of atherosclerosis and protects against acute coronary syndrome and ischemia-reperfusion injury [51].

Animal studies showed that a fiber-rich diet changes the gut microbiota, thus contributing to the prevention of cardiovascular diseases. The interpolation of these findings to dietary intervention in humans might be a cost-effective approach to prevent hypertension [52]. 

Lifestyle changes are essential in the non-pharmacological management of hypertension, including a healthy diet with low-lipid and high-fiber content (rich in fruits and vegetables). In addition to proper nutrition, dietary supplements are also important due to their vitamin and mineral content [53,54].

In Western European countries, the average dietary fiber intake is around 15 g/day, which represents about half of the recommended amount. The consumption of ultra-processed food might be related to the increased incidence of hypertension [54]. 

Regarding lipid profile, lower serum cholesterol levels were found in hypertensive patients with cognitive dysfunction; the difference is not quite significant, probably due to their medication and more restrictive diet. These results are in close connection with the significantly lower daily cholesterol intake observed in this subgroup compared to those with normal cognitive status. Previous studies reported that increased serum cholesterol levels in a person’s midlife increased the risk of cognitive impairment in late life, but high total cholesterol in late life was not associated with mild cognitive decline or dementia [3]. Increasing the quantity of fibers in the diet of the hypertensive patients could be beneficial for cholesterol homeostasis [55].

The negative correlation between triglyceridemia and PUFA intake is similar to the outcome of certain previous studies in the literature, but the relationship is controversial and the results depend on the type of PUFA [56,57].

Significantly higher levels of microalbuminuria are present in the subgroup with impaired cognitive function, which can be explained by the manifestation of microvasculopathy of the affected organs (brain and kidneys). In subjects with DM as a comorbidity, microalbuminuria can also occur due to diabetic nephropathy [58]. Increasing the daily water intake would be an appropriate change in lifestyle habits of hypertensive patients to improve kidney function.

The incidence of high body weight and obesity was high in the studied group, especially in the hypertensive subgroup with cognitive dysfunction. Calorie intake exceeded the recommended levels in over half of the studied subjects; therefore, professional nutritional advice would be appropriate for these patients to improve these values. Reducing body weight can lower the risk of impaired carbohydrate and lipid metabolism in hypertensive patients as obesity is a risk factor for dyslipidemia and DM, and it increases the risk of developing hypertension [59]. Our study showed a frequent association of hypercaloric diet and obesity. A total of 55% of the subjects were on the hypercaloric diet based on their reported dietary habits. The authors consider that raising awareness about healthy nutrition can be a way to prevent complications.

Chronic inflammation is common in chronic diseases, such as obesity, hypertension and DM [60,61]; we observed slightly higher levels of inflammatory markers in hypertensive patients with cognitive dysfunction. 

The limitations of this study are the small number of enrolled subjects due to the lockdown and COVID-19 restrictions, the assessment of only a few selected inflammatory markers and the reduced number of studied minerals. Unfortunately, the study was seriously delayed in the context of the coronavirus pandemic compared to the original schedule due to the closure of the hosting clinical unit for several months, and the unfavorable epidemiological situation caused serious restrictions in the number of the enrolled patients. Another limitation refers to the accuracy of the nutritional survey data, which were based on a questionnaire completed by the patients, so the provided information might be prone to certain distortions.

The importance and novelty of the study is derived from the complex evaluation of the patients, corroborating the data of the nutritional survey with laboratory tests. Several parameters included in this study are not used as a routine, such as serum zinc, cystatin C and hsCRP measurement. Data provided by our research can contribute to better management of hypertensive patients, revealing improper dietary habits, deficiencies and cardiovascular risk factors. 

The study has several practical implications, especially regarding laboratory testing and nutrition advice given to hypertensive patients. More complex investigations should be carried out in patients with chronic cardiovascular and metabolic diseases, including the assessment of vitamin and trace element levels. Fiber intake and daily liquid consumption should be increased in several cases; the supplementation of vitamins and minerals is recommended in cases of deficiency.

## 5. Conclusions

The higher intake of antioxidant vitamins, zinc, magnesium and carotenoids can be recommended for hypertensive patients, especially those with impaired glucose metabolism, carotenoid, zinc and magnesium deficiencies. Increased water intake, fiber-rich, low-calorie and low-cholesterol diet would be appropriate for the subjects. 

Nutritional surveys should be administered to patients with chronic diseases and a wider range of laboratory tests should be carried out to obtain necessary data to evaluate the risk factors for complications, thus enabling adequate prevention. 

## Figures and Tables

**Table 1:** Comparison of BMI, duration of hypertension and laboratory parameters in the studied patient groups. Statistically significant differences are marked with asterisks.

Parameter	Unit	Hypertensives with Cognitive Dysfunction	Hypertensives without Cognitive Dysfunction	Statistical Significance
**Duration of** **hypertension**	years	19.08 ± 12.27	17.08 ± 12.19	*p* = 0.574
**BMI**	kg/m^2^	32.61 ± 7.43	27.82 ± 4.36	*p* = 0.009 *
**Glycemia**	mmol/L	5.99 ± 1.26	5.53 ± 1.02	*p* = 0.159
**Cholesterol**	mmol/L	4.48 ± 1.03	5.06 ± 1.15	*p* = 0.071
**Triglycerides**	mmol/L	1.53 ± 0.89	1.58 ± 0.68	*p* = 0.813
**HDL**	mg/dL	44.76 ± 13.52	50.14 ± 12.60	*p* = 0.152
**LDL**	mmol/L	2.61 ±1.11	3.05 ± 1.03	*p* = 0.156
**Uric acid**	µmol/L	303.76 ± 78.85	278.84 ± 96.95	*p* = 0.324
**Creatinine**	mg/dL	0.99 ±0.22	0.99 ± 0.26	*p* = 0.940
**Cystatin C**	mg/L	1.11 ± 0.34	1.12 ± 0.24	*p* = 0.955
**Zinc**	µmol/L	10.21 ± 1.58	9.99 ± 1.65	*p* = 0.631
**HsCRP**	mg/L	2.94 ± 3.76	1.56 ± 1.82	*p* = 0.107
**ESR**	mm/h	14.36 ± 8.89	10.76 ± 7.49	*p* = 0.128
**Fibrinogen**	g/dL	3.92 ± 1.08	3.39 ± 0.74	*p* = 0.066

* (*p* < 0.05): there is a significant statistically difference.

**Table 2:** Comparison of daily dietary nutrient intake in hypertensive patients with/without cognitive dysfunction. Statistically significant differences are marked with asterisks.

Dietary Intake/Day	Unit	Hypertensives with Cognitive Dysfunction	Hypertensives without Cognitive Dysfunction	Statistical Significance
**Energy**	kcal/day	2157.39 ± 974.80	2296.41 ± 860.65	*p* = 0.615
**Water**	mL/day	1423.00 ± 457.16	1620.59 ± 520.55	*p =* 0.183
**Protein**	g/day	90.43 ± 39.63	96.18 ± 41.60	*p =* 0.637
**Fat**	g/day	94.43 ± 46.83	103.45 ± 43.24	*p =* 0.506
**Carbohydrates**	g/day	215.39 ± 82.88	232.09 ± 81.06	*p =* 0.498
**Dietary fibers**	g/day	26.70 ± 9.53	31.45 ± 11.70	*p =* 0.141
**PUFA**	g/day	19.43 ± 12.12	23.18 ± 10.35	*p =* 0.272
**Cholesterol**	mg/day	288.61 ± 139.08	409.27 ± 198.69	*p =* 0.022 *
**Alcohol**	g/day	1.83 ± 5.01	3.05 ± 4.45	*p =* 0.393

* (*p* < 0.05): there is a significant statistically difference.

**Table 3:** Dietary supplements daily intake in hypertensive patients.

Dietary Intake/Day	Unit	Hypertensives with Cognitive Dysfunction	Hypertensives without Cognitive Dysfunction	Statistical Significance
**Vitamin A**	µg/day	1606.17 ± 617.77	1909.45 ± 796.24	*p =* 0.160
**Carotene**	mg/day	6.09 ± 2.43	7.18 ± 3.07	*p =* 0.190
**Vitamin E**	mg/day	22.74 ± 14.16	26.09 ± 12.03	*p =* 0.398
**Vitamin B_1_**	mg/day	1.17 ± 0.65	1.27 ± 0.77	*p =* 0.643
**Vitamin B_2_**	mg/day	1.74 ± 0.81	1.95 ± 0.79	*p =* 0.370
**Vitamin B_6_**	mg/day	1.78 ± 0.78	2.05 ± 0.79	*p =* 0.271
**Folic acid**	µg/day	298.09 ± 141.77	362.68 ± 150.52	*p =* 0.145
**Vitamin C**	mg/day	193.52 ± 98.64	209.68 ± 106.60	*p =* 0.600
**Sodium**	mg/day	1767.57 ± 952.17	1797.18 ± 762.79	*p =* 0.909
**Potassium**	mg/day	3389.65 ± 1266.35	3932.41 ± 1493.86	*p =* 0.195
**Calcium**	mg/day	1399.96 ± 1188.14	1196.77 ± 555.03	*p =* 0.470
**Magnesium**	mg/day	323.65 ± 126.61	419.45 ± 159.56	*p =* 0.032 *
**Phosphorus**	mg/day	1376.17 ± 614.93	1615.09 ± 634.38	*p =* 0.206
**Iron**	mg/day	13.35 ± 5.21	15.91 ± 6.06	*p =* 0.135
**Zinc**	mg/day	13.52 ± 6.04	14.00 ± 6.10	*p =* 0.793

* (*p* < 0.05): there is a significant statistically difference.

**Table 4:** Reference ranges of the measured laboratory parameters.

Parameter	Unit	Reference Range Male Patients	Reference Range Female Patients	Notes
Glycemia	mmol/L	3.5–5.6	3.5–5.6	For non-diabetics
Cholesterol	mmol/L	3.9–5.2	3.9–5.2	In high risk group < 4.6
Triglycerides	mmol/L	0.45–1.81	0.40–1.52	
HDL	mg/dL	>40	>50	
LDL	mmol/L	<2.6	<2.6	
Uric acid	µmol/L	240–510	160–430	
Creatinine	mg/dL	0.74–1.35	0.59–1.04	
Cystatin C	mg/L	0.7–1.10	0.57–1.03	
Zinc	µmol/L	10.4–16.4	10.4–16.4	
HsCRP	mg/L	<1	<1	
ESR	mm/h	5–15	5–17	
Fibrinogen	g/dL	2.0–4.0	2.0–4.0	
Microalbuminuria	mg/24 h	<30	<30	

**Table 5:** Recommended daily intake of nutrients for adults depending on health status.

Dietary Intake/Day	Units	Recommended/Acceptable for Healthy Subjects	Special Recommendations (Hypertension/Overweight)
Energy	kcal/day	30–35 kcal/kg	20–25 kcal/kg
Water	mL/day	30–35 mL/kg	
Alcohol	g/day	acceptable moderate intake: 20–30 g/day in males 10–20 g/day in females	No alcohol intake
Protein	g/day	12–20% of total calories (0.8–2.0 g/kg) 46–56 g/day	15–18% of total calories (1.0–1.8 g/kg)
Fat	g/day	20–35% of total calories (<10% saturated) 44–77 g/day	20–30% of total calories (<6% saturated)
Carbohydrates	g/day	45–65% of total calories 174–194 g/day	45–55% of total calories
Dietary fibers	g/day	30 g/day	
PUFA	g/day	6–11% of total calories 1.1–1.6 g/day	
Cholesterol	mg/day	<300 mg/day	<150 mg/day

**Table 6:** Recommended daily intake of dietary supplements for adults depending on health status.

Dietary Intake/Day	Units	Recommended/Acceptable for Healthy Subjects	Special Recommendations (Hypertension/Overweight)
Vitamin A	µg/day	900 µg/day in males 700 µg/day in females	
Carotene	mg/day	12–15 mg/day	
Vitamin E	mg/day	12–20 mg/day	
Vitamin B_1_	mg/day	1–2 mg/day	
Vitamin B_2_	mg/day	1.1–1.2 mg/day	
Vitamin B_6_	mg/day	2 mg/day	
Folic acid	µg/day	400 µg/day	
Vitamin C	mg/day	>120 mg/day	
Sodium	mg/day	5–6 g/day	1.5–3 g/day
Potassium	mg/day	4600–4800 mg/day	
Calcium	mg/day	850–1500 mg/day	
Magnesium	mg/day	400–500 mg/day	
Phosphorus	mg/day	800–1000 mg/day	
Iron	mg/day	10–18 mg/day	
Zinc	mg/day	12–15 mg/day	

## Data Availability

Reported results can be found on the personal drive and can be provided on request by the study coordinator.

## Abbreviations

BMI	body mass index
DM	diabetes mellitus
ESR	erythrocyte sedimentation rate
HDL	high density lipoproteins
hsCRP	high sensitivity C reactive protein
LDL	low density lipoproteins
MOCA	Montreal Cognitive Assessment
NIH	National Institutes of Health
PUFA	polyunsaturated fatty acids
